# Sustainable Setups for the Biocatalytic Production and Scale-Up of Panthenyl Monoacyl Esters under Solvent-Free Conditions

**DOI:** 10.1021/acssuschemeng.3c00266

**Published:** 2023-03-22

**Authors:** Susana Nieto, Juana M. Bernal, Rocio Villa, Eduardo Garcia-Verdugo, Antonio Donaire, Pedro Lozano

**Affiliations:** † Departamento de Bioquimica y Biologia Molecular B e Inmunologia. Facultad de Quimica, Campus de Espinardo, Universidad de Murcia, E-30100 Murcia, Spain; ‡ Department of Biotechnology, Delft University of Technology, Building 58 Van der Maasweg 9, 2629 HZ Delft, The Netherlands; § Departamento de Quimica Organica e Inorganica, Universidad Jaume I, E-12071 Castellon, Spain; ∥ Departamento de Quimica Inorganica. Facultad de Quimica, Campus de Espinardo, Universidad de Murcia, E-30100 Murcia, Spain

**Keywords:** biocatalysis, panthenol esters, solvent-free, scaling-up, sustainable processes

## Abstract

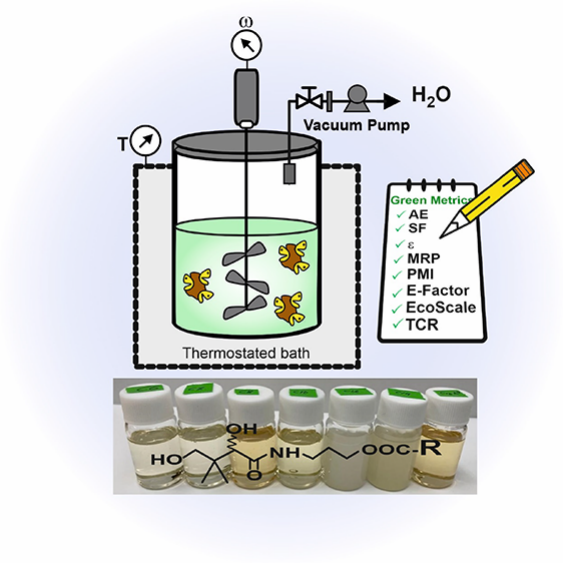

A sustainable scaling-up process for the biocatalytic production of new bioactive provitamin-B_5_ monoacyl esters has been demonstrated. A solvent-free reaction protocol, based on the formation of eutectic mixtures between neat substrates, renders highly efficient direct esterification of free fatty acids (*i.e*., from C_6_ to C_18_ alkyl-chain length) with panthenol catalyzed by lipase. The scale-up from 0.5 to 500 g was evaluated by means of using several reaction systems (*i.e.*, ultrasound assistance, orbital shaking, rotary evaporator, and mechanical stirring coupled to vacuum). For all reactor systems, the yield in panthenyl monoacyl esters was improved by increasing the length of the alkyl chain of the fatty acid (*i.e.*, from 63% yield for panthenyl butyrate to 83% yield for panthenyl myristate). The best results (87–95% product yield, for all cases) were obtained upon a scale-up (50–500 g size) and when a vacuum system was coupled to the biocatalytic reaction unit. Under the optimized conditions, a 5-fold reduction of the amount of biocatalysts with respect to reactors without vacuum was achieved. The recovery and reuse of the immobilized enzyme for five operation cycles were also demonstrated. Finally, different metrics have been applied to assess the greenness of the solvent-free biocatalytic synthesis of panthenyl monoesters here reported.

## Abstract 2

Solvent-free biocatalytic synthesis of bioactive panthenyl monoacyl esters is the ideal sustainable approach for scaling-up, as results of high product yields and excellent green metrics analyses.

## Introduction

Cosmetics is a growing field with a worldwide market of around US$380.2 billion in 2019 and is expected to continue increasing in the coming years.^1^ This field is particularly sensitive to the sustainable synthesis of its ingredients and formulas, imposed by global regulations^2,3^ and the consumer’s demands.^4^ In addition, the high competitiveness boosts the research and constant updating of the products to gain the dominant position. In this sense, a current trend is focused on the so-called “cosmeceuticals”, products that combine both cosmetics benefits and bioactive effects on health.^5^

Panthenol (2,4-dihydroxy-*N*-(3-hydroxypropyl)-3,3-dimethylbutanamide) is a usual ingredient in cosmetics due to its moisturizing capacity and its bioactive properties. In these regards, as the precursor of vitamin B_5_, panthenol can also be considered a cosmeceutical ingredient because of its beneficial health effects (*i.e.*, promotes lipid synthesis, fibroblast proliferation, and wound healing, has anti-inflammatory and anti-pruritic roles, fights against stress-induced radicals, etc.), being highly convenient for sensitive skin health treatments.^6−9^

To improve the dermal absorption and long-lasting effects of panthenol, the three hydroxyl free groups are usually blocked by acylation. In this context, the selective tailoring of panthenol with different alkyl-chain length fatty acids to achieve novel cosmeceuticals with on-demand physical–chemical properties is a highly attractive goal from an industrial perspective. In addition, the amphiphilic nature of these derivatives can also lead to additional surfactant activity, which is of high interest for the preparation of cosmetic formulations. However, the synthesis of panthenyl monoacyl esters by chemical or biocatalytic approaches has been seldom reported. The presence of three different hydroxyl groups hampers the development of selective and high-yield synthetic protocols to obtain panthenol derivatives. In general, selective synthesis of monoester panthenol derivatives requires classical synthetic protocol based on protection and deprotection of hydroxyl groups, in contradiction with the eighth principle of green chemistry. For instance, the synthesis of panthenyl docosahexaenoate has been reported by this approach to only afford, despite the protection/deprotection, a modest 54% monoester yield.^10^ On the other hand, the reaction of free panthenol with acetic anhydride in the presence of a heterogeneous catalyst based on dimethylaminopyridine groups at 80 °C leads to the esterification of all hydroxyl groups of panthenol with a good yield of panthenyl triacetate (up to 80%) as the only product ruling out the selective monoesterification.^11^ Alternatively, the use of lipases has allowed a more selective esterification of the two primary hydroxyl groups of panthenol. Thus, the biocatalytic synthesis of both panthenyl monoacyl ester (PME) and panthenyl diacyl ester (PDE) was carried out by transesterification reactions using short aliphatic esters, such as isopropyl acetate^12^ or ethyl acrylate,^13^ as acyl donors using volatile organic solvents (*i.e.*, acetonitrile, acetone, *tert*-butyl methyl ether, etc.) as reaction media. By this approach, a final mixture with a variable composition of both the PME and PDE products was obtained (60–96% PME and 4–40% PDE) for the best conditions.

As mentioned above, sustainability plays a fundamental role in today’s cosmetic industry. Indeed, there is an increasing demand for greener synthetic methods, allowing efficient and clean approaches at the industrial scale (*i.e.*, biocatalysis, solvent-free approaches, recovery and reuse of solvents, simple and straightforward purification protocols, etc.).^14,15^ At the industrial scale, the selectivity of catalytic transformations and the easy separation of pure products are two key axes that determine the economy and sustainability of chemical processes. From the green point of view (*i.e.*, atom economy, waste generation, etc.), the direct esterification between carboxylic acids and alcohols is an outstanding approach for ester synthesis because of the use of non-derivatized natural substrates that avoid the generation of byproducts, which favor the easy separation of ester products and simplifying any further isolation and purification step of products.^16^ In these regards, it is widely recognized that ionic liquids and supercritical fluids lead to amazing synergies with biocatalysts that not only improve the catalytic efficiency but also allow simple and smart strategies for product isolation and simultaneous full recovery and reuse of the enzyme and the reaction media.^17^ Indeed, a sustainable approach for the biocatalytic synthesis of different PMEs (*i.e.*, hexanoate, laurate, palmitate, etc.) was successfully carried out by the direct esterification of the corresponding free fatty acid with panthenol in different sponge-like ionic liquids (*i.e.*, 1-dodecyl-3-methylimidazolium tetrafluoroborate [C_12_mim][BF_4_], etc.) with up to 90% conversion and 100% selectivity of PME. Furthermore, the sponge-like behavior enabled the easy recovery of both the biocatalyst and IL for further reuse, preventing waste generation.^18^ However, from an industrial and sustainable point of view, a synthetic protocol to obtain panthenol derivatives under solvent-free conditions will provide a simplification of the downstream processing and a reduction of the cost and detrimental issues associated with the purification of products and solvent recovery and significantly decreasing the waste generation.^19^ For the case of solid substrates, such as panthenol, the formation of eutectic mixture (or deep eutectic solvents (DESs)) liquid at room temperature^20−22^ between the neat substrates of the reaction opens a new pathway for the development of solvent-free reactions.^23^ In this context, we have been pioneers in the development of deep eutectic mixtures of panthenol and free fatty acids as excellent reaction media for the biocatalytic synthesis of PMEs (*i.e.*, up to 83% yield for panthenyl monolaurate).^18,24^ The interest in using lipases as biocatalysts in this approach is beyond their excellent performance to achieve the selective esterification of the hydroxyl groups of panthenol in just one step and with higher efficiency. Lipases, as natural catalysts, are also biodegradable and confer the “natural” label to the products they synthesize in agreement with the current sustainable agenda of the cosmetic industry.^25,26^

**Figure 1:**
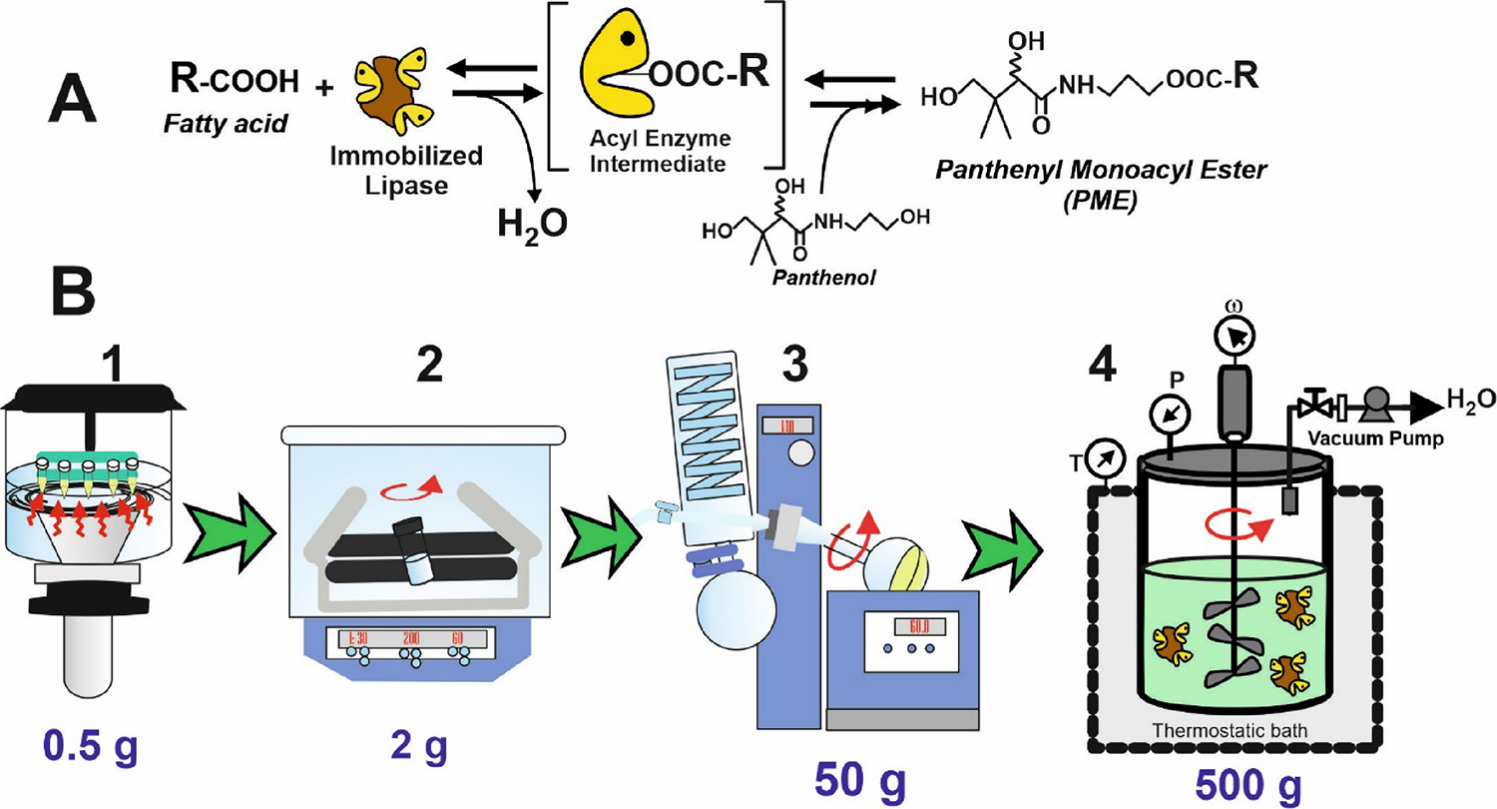
(A) General scheme of the lipase-catalyzed synthesis of PME by direct esterification of free carboxylic acids with panthenol. (B) Different reaction setups evaluated for scale-up of the biocatalytic synthesis of PME under solvent-free conditions (**1**, ultrasonic assistance; **2**, orbital shaker; **3**, rotary evaporator with vacuum; **4**, mechanically stirred reactor with vacuum).

One of the main drawbacks of the implementation of eutectic mixtures as the biocatalytic reaction medium is their high viscosity. The addition of water can partially reduce the DES viscosity, enabling their use even under flow conditions. For instance, it has been reported how the addition of water (up to 15% v/v) on DESs based on choline chloride/glycerol (1/2) leads to solvent mixtures with viscosities below 25 mPa·s that enable efficient enzymatic reactions in flow microreactor devices.^27^ In any case, it is highly important to remove the water from the enzyme microenvironment for this solvent-free approach because this byproduct can shift the reversible equilibria toward the hydrolysis of the product with a detrimental impact on the final yield (see Figure 1A).^17,18^ Alternatively, different approaches can be used to improve the mass transfer overcoming the viscosity issues. Among them, the ultrasonic assistance has been shown as a highly efficient tool for the biocatalytic esterification of free fatty acids (*i.e.*, lauric acid) with polyhydroxyl compounds (*i.e.*, xylitol) under solvent-free conditions. Thus, the use of ultrasounds was key to overcoming the mutual immiscibility of fatty acids and xylitol substrates and the semisolid character of the initial reaction mixtures, enabling the transport of substrate molecules to the enzyme catalytic site for an efficient synthesis of xylityl monoacyl and diacyl esters as the main products with a 96% yield.^28^

Here, this work reports on the evaluation of different green protocols, *i.e.*, ultrasound assistance, orbital shaking, rotary evaporator, and 500 g stirred reactor (see Figure 1B) for the biosynthesis and scale-up of PME synthesis under solvent-free conditions. The influence of the alkyl-chain length of the fatty acid, the amount of the enzyme, as well as the role of different setups for mixing that can be coupled to a vacuum system have been studied, being selected the best operational protocol and operation unit for scaling-up, with regard to the efficiency on yield, sustainability, and environmental impact as determined by using different green metric parameters, like the PMI, the E-factor, and the EcoScale tool.

## Experimental Section

### Chemicals

Immobilized *Candida antarctica* lipase B (Novozym 435, N435) was provided by Novozymes S.A. (Spain). All free fatty acids were purchased from Sigma-Aldrich, with a purity ranging from 96 to 99.5%: butyric acid (C_4_), pentanoic acid (C_5_), hexanoic acid (C_6_), heptanoic acid (C_7_), octanoic acid (C_8_), decanoic acid (C_10_), lauric acid (C_12_), myristic acid (C_14_), and oleic acid (Δ^9^ C_18_). The desiccant agent molecular sieve 13× (MS13×; 270 mg H_2_O/g adsorption capacity) was also provided by Sigma-Aldrich-Fluka (Madrid, Spain).

### Biocatalytic Esterification Reactions

A gradual scaling-up of the reaction mixture was performed using different setups. The overall mass ranged from 0.2 g in the ultrasound system, 2 g for orbital shaking, 10–66 g for the rotary evaporator, and 300–500 g for mechanical stirring coupled to a vacuum system.

The previously described procedure^18^ to obtain the eutectic mixture and perform the reaction was followed. Briefly, mixtures of selected molar ratios of panthenol and the corresponding free fatty acid (FFA) were prepared. Once the eutectic mixtures were obtained, the reaction started with the addition of the biocatalysts Novozym 435, ranging from 2.5 to 12.5 mg/mmol overall mass, and the desiccant agent MS 13× (12.5 mg/mmol overall mass) and posterior incubation at 60 °C for a range of 2–8 h according to the reactor system used. The conditions for the ultrasound-assistance setup were 40 °C, 70% amplitude, and 2 h reaction time, as previously reported.^28^

To perform kinetic profiles, aliquots of 20 μL were withdrawn at regular intervals and suspended in 980 μL of acetonitrile/methanol/H_2_O (35:15:50 v/v/v) for further analysis in HPLC.

### Recovery and Reuse of Biocatalysts

To demonstrate the stability of N435, the enzyme was recovered and reused in several continuous cycles of synthesis performed in the rotary evaporator. Briefly, after each biocatalytic synthesis, the reaction media was collected and centrifuged at 6000 rpm for 10 min at 25 °C, resulting in the precipitation of the immobilized enzyme particles. Then, the biocatalyst was separated by simple decantation of the liquid media and stored at 4 °C until further reuse. To perform the reuse cycles, a fresh eutectic mixture of substrates was added to the recovered enzyme, which proceeded with a new biocatalytic esterification cycle for 8 h at 60 °C. Alternatively, the immobilized enzyme recovered from the first cycle was also stored for 2 months at 4 °C, and the residual activity was determined after this period.

### HPLC-ELSD Analysis

The separation and identification of the substrates and products were performed in an RP-C18 column LiChroCART-LiChrospher (100 m × 0.25 mm × 5 μm, Merck, USA) on a Shimadzu LC-20 HPLC (Shimadzu Europe, Germany) equipped with an evaporative light scattering dispersive detector (ELSD-LT II, Shimadzu), operating at 335 KPa, 40 °C, gain 4.

The elution conditions were 50 °C oven temperature and 1 mL min^–1^ flow rate of a mixture of the solvents: A, acetonitrile; B, methanol; C, water. When using FFAs with a chain length ≤7C in the esterification reaction, the following gradient of elution was used: 0.01 min (15:15:70 v/v), 15–25 min (75:15:10 v/v), and 26–30 min (15:15:70 v/v). For FFAs with longer alkyl chains, more hydrophobic initial conditions were used: 0.01 min (35:15:50 v/v), 10 min (75:15:10 v/v), 10–18 min (75:15:10 v/v), and 19–30 min (35:15:50 v/v). Panthenol and panthenyl ester products were quantified by an ELSD detector. Peak retention times (min) were as follows: panthenol, 2.5; panthenyl monobutyrate, 8.7; panthenyl dibutyrate, 13.4; panthenyl monopentanoate, 10.3; panthenyl dipentanoate, 15.6; panthenyl monohexanoate, 11.9; panthenyl dihexanoate, 17.6; panthenyl monoheptanoate, 13.3; panthenyl diheptanoate, 19.2; panthenyl monooctanoate, 6.0; panthenyl dioctanoate, 16.0; panthenyl monodecanoate, 9.2; panthenyl didecanoate, 19.3; panthenyl monolaurate, 13.1; panthenyl dilaurate, 21.1; panthenyl monomyristate, 16.7; panthenyl monooleate, 20.4. Free carboxylic acids were detected by a DAD system at 205 nm. Peak retention times (min) were as follows: butyric acid, 3.3; valeric acid, 4.8; hexanoic acid, 7.5; heptanoic acid, 9.9; octanoic acid, 3.9; decanoic acid, 6.2; lauric acid, 10.1; myristic acid, 16.2; oleic acid, 18.2.

### ^1^H NMR and ^13^C NMR Analyses

Nuclear magnetic resonance spectroscopy was performed using a Bruker Advance 600 MHz spectrometer equipped with triple gradient TXI (^1^H/^13^C/^15^N) and broadband (^13^C) probes. The esterification of hexanoic acid with panthenol performed in the rotary evaporator was selected as the reaction model. An aliquot of 50 μL of the sample was dissolved in 500 μL of DMSO-δ_6_ and analyzed by NMR at room temperature. The assignment of the protons was achieved by analogy to the 2D homonuclear (COSY, TOCSY, and NOESY) and heteronuclear (^1^H–^13^C HMQC) experiments previously performed.^18^ DOSY experiments were also performed to discern and quantify among panthenol, monoester, and diester species (see the SI, Section 2). Signals of panthenyl hexanoate were at the same chemical shifts as panthenol or hexanoic acid except explicitly indicated.

^1^H NMR δ (ppm; E: panthenyl in ester, H: hexanoate in ester): 0.78 (s, 3H, H_3_E); 0.79 (s, 3H, H_3_E); 0.84 (t, 3H, H_6_H); 1.24 (m, 2H, H_4_-H_5_H); 1.50–1.54 (m, 3, H_1_, E-hydroxypropyl, and H_3_H); 1.71 (m, 1H, H_1_E-hydroxypropyl); 2.26 (t, 2H, H_2_H); 3.09 (m, 1H, H_1_E-hydroxypropyl); 3.16 (m, H, H_1_E-hydroxypropyl); 3.29 (m, 1H, H_4_E-hydroxypropyl); 3.40 (t, 2H, H_3_E); 3.70 (s, 1H, H_2_E); 3.99 (t, 1H, H_3_E-hydroxypropyl). ^13^C NMR δ (ppm, P: panthenol; E: panthenyl in ester, H: hexanoate in ester): 15.2 (C_6_H); 21.8 (C_3_P); 22.4 (C_3_P); 23.2 (C_5_H); 25.6 (C_3_H); 29.9 (C_2_E-hydroxypropyl); 32.1 (C_4_H); 33.8 (C_4_H); 33.8 (C_2_P-hydroxypropyl); 34.9 (C_2_H); 36.5 (C_1_E-hydroxypropyl); 37.0 (C_1_P-hydroxypropyl); 40.5 (C_3_E); 60.9 (C_3_P-hydroxypropyl); 63.2 (C_3_E-hydroxypropyl); 69.6 (C_4_E); 76.6 (C_2_E); 174.4 (C carbonyl, P); 174.5 (C carbonyl, E).

### Physical–Chemical Characterization of Panthenyl Esters

Reaction mixtures of the biocatalytic esterification performed in the rotary evaporator setup were selected to determine the pH, viscosity, and dry residue parameters.

The pH was evaluated using a pH-meter (SensION+, Germany), and the viscosities were measured using a P-Selecta Viscometer (ST-2020R, Spain), with spindles R4* and R7** depending on the viscosity value and an angular speed of 20 rpm. The dry residue was quantified with thermobalance equipment (Kern DBS, Germany) using a constant temperature of 120 °C.

### Tools for PMI and EcoScale Calculation

The PMI value was calculated using the ACS PMI Calculator (available at https://www.acs.org/content/acs/en/greenchemistry/research-innovation/tools-for-green-chemistry.html).^29^ Also, the ecological–economic impact of the strategy was evaluated using the EcoScale tool (available at http://ecoscale.cheminfo.org/calculator).^30^

## Results

### Sustainability of the Biocatalytic Synthesis of PMEs by Esterification

The biocatalytic esterification of panthenol with hexanoic acid in a solvent-free reaction medium was selected as a benchmark reaction to evaluate the effect of the molar ratio of panthenol/fatty acid. It should be noted that for all the molar ratios assayed, a eutectic mixture was formed by just heating and stirring the substrate mixtures up to 70 °C for 1 h. The monitoring of biocatalytic synthesis of the corresponding esters was carried out by HPLC analysis. Both the reaction conversion (amount of panthenol converted to ester products) and selectivity (abundance of PMEs within the products) were highly dependent on the molar ratio of panthenol:hexanoic acid used (see Table 1).

**Table 1:** Effect of the Panthenol (P):Hexanoic Acid (HA) Molar Ratio on the Biocatalytic Synthesis of Panthenyl Monohexanoate by Direct Esterification under Solvent-Free Conditions in a 0.5 g Orbital Shaker for 6 h at 60 °C Using 12.5 mg N435/mmol Overall Mass

entry	P:HA (mol:mol)	conv (%)^a^	sel (%)^b^
1	2:1	32	94
2	1:1	67	91
3	1:2	87	67
4	1:3	100	49

a Conversion: percentage of panthenol converted to ester products.

b Selectivity: percentage of PME within the total ester products.

According to the data in Table 1, by increasing the carboxylic acid content with respect to panthenol in the substrate mixture, the transformation of panthenol to ester products was increased up to 100%. Nevertheless, a concomitant loss in selectivity toward monoacyl ester synthesis was also observed. Considering that the catalytic mechanism of the lipase-catalyzed esterification reaction is mediated through an acyl-enzyme intermediate (see Figure 1A), the increase of the acyl donor (hexanoic acid) concentration with respect to the nucleophile acceptor (panthenol) improves the enzymatic esterification conversion. However, due to the presence of three hydroxyl groups on the panthenol molecule, the excess of the acyl donor also leads to the multi-esterification of the polyalcohol with the identification of panthenyl diesters that decreases the selectivity of the reaction, though the panthenyl trihexanoate product was never detected.

On the contrary, by increasing the amount of panthenol with respect to the carboxylic acid content (2:1 ratio), the selectivity improved up to 94% at the expense of the conversion of panthenol to panthenyl ester products, which was reduced by half with respect to the 1:1 substrate molar ratio (see entries 1 and 2, Table 1). It should be noted that the higher content of panthenol involves an increase in the viscosity of the reaction medium, which may negatively affect the mass-transfer phenomena from the bulk media to the enzyme microenvironment, reducing the esterification conversion.

Despite conversion being an important criterion to optimize the reaction efficiency, it is equally relevant to consider the selectivity and other green parameters, especially when the aim is to implement the reaction at an industrial scale. Thus, the yield (ε), atom economy (AE), stoichiometric factor (SF), material recovery parameter (MRP), and reaction mass efficiency (RME) parameters were used as simple green metrics to evaluate simultaneously the efficiency and environmental impact of the process in the selective synthesis of panthenyl monoesters. Table 2 shows the equations used for the quantification of all the green metric parameters used in this work.

**Table 2:** Green Metric Parameters Used in This Work^31^

parameter	abbrev.	calculation	
atom economy	AE	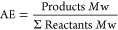	(1)
stoichiometric factor	SF	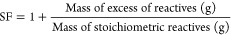	(2)
yield of panthenol monoesters^a^	ε	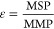	(3)
material recovery parameter^b^	MRP	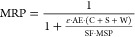	(4)
reaction mass efficiency	RME	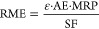	(5)
process mass intensification	PMI	PMI	(6)
E-factor	E-factor	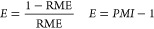	(7)
total carbon dioxide release	TCR	TCR = ( PMI organic × 2.3 + PMI water × 0.63)	(8)

a MSP, mass of synthesized monoester products; MMP, maximum mass of synthesized products.

b C, catalyst (g); S, substrates (g); W, wastes (g).

The AE, a parameter introduced by Trost,^32,33^ affords the quantification of the atoms incorporated into the final product and the evaluation of the amount of waste produced in a reaction. The SF refers to the molar ratio of substrates of the reaction system and permits to perform calculations when using one or more reactants in excess with respect to a limiting one. Since SF > 1 for any non-stoichiometric reaction,^31^ the inverse of SF (1/SF) is commonly used to normalize the value (0 < 1/SF < 1) for green metric studies. The ε parameter provides an insight into the reactivity of substrates and quantifies the selective (bio)catalytic productivity of the reaction, being determined from panthenol and its ester derivative concentrations in all cases. Meanwhile, AE, SF, and ε parameters only consider the substrates for calculation, and the contribution of other reaction elements like the solvent (S), catalysts (C), or wastes (W) is accounted for by MRP and RME parameters. The MRP parameter is a term introduced by Andraos^34^ that points to the loss of auxiliary materials not recovered along the reaction and downstream steps. The RME parameter contains all the above metrics providing an overall view of the process sustainability. In this respect, RME outstands as the most informative green metric parameter and is also correlated with Sheldon’s environmental factor (E-factor).^35,36^

**Figure 2:**
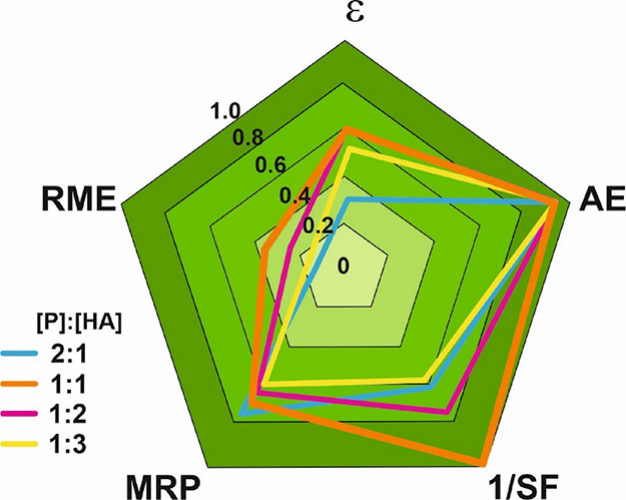
Radial pentagon diagram of the green metrics parameters determined at different panthenol (P) and hexanoic acid (HA) molar ratios in the biocatalytic synthesis of panthenyl monohexanoate (Table 1). P:HA molar ratios, 2:1 mol/mol (blue); 1:1 mol/mol (orange); 1:2 mol/mol (purple); 1:3 mol/mol (yellow).

Altogether, the combination of these green metrics affords the evaluation of a reaction considering the efficiency and the harnessing of substrates and auxiliary materials. Figure 2 shows a radial pentagon diagram as proposed by Andraos to graphically illustrate the results of the green metrics analysis for the selective synthesis of panthenyl monohexanoate at each molar ratio of substrates used in Table 2.^34^ Assuming a value of 1 for each pentagon radius as the ideal condition for a sustainable process, the closer the value of the different metrics, the greener the process.^31^

In this work, the C (catalyst) and S (solvent) terms in MRP calculation (eq 4) were considered null because this biocatalytic esterification proceeded with full recovery and reuse of the biocatalysts and under solvent-free conditions. In the same way, the waste (W) term accounts for the contribution of the byproduct water and non-reacted substrates to compare the efficiency of each molar ratio of substrates, although the excess of substrates is not a waste *per se* as they are also cosmetic ingredients and do not need to be removed.

Taking into account that the diagram of Figure 2 compares the direct esterification approach at different molar ratios of substrates, all cases share the same high value for the AE parameter as results of the negligible contribution of water as the byproduct. According to the diagram, the lower ε (0.3) was obtained for the higher P:HA molar ratio (2:1), while the other conditions showed a similar ε value (0.5–0.6). This diagram shows that an increase in fatty acid content with respect to panthenol does not lead to a higher monoester yield, as suggested by Table 1. Thus, the equimolar ratio of substrates resulted in the most balanced pentagon in the figure and with the best scores for almost all metrics, including the RME parameter (0.4). At this equimolar ratio of substrates, the biocatalytic synthesis of PME was achieved with 67% yield and 91% selectivity toward the monoester target product. The NMR analyses confirmed that a primary hydroxyl group of the C3 hydroxypropyl position of panthenol is involved in the ester linkage in the vast majority of the monoester products (77%), as results of the higher nucleophilicity of this primary alcohol (see Figure S1).

Unlike other esterification reactions when using monohydroxyl compounds (*e.g.*, menthol^37^) in solvent-free systems, the selective monoesterification of panthenol, having three hydroxyl groups, compels to the careful selection of the molar ratio of substrates to obtain an equilibrium between the best esterification yield and the higher selectivity. These results clearly point to the suitability of the proposed biocatalytic approach for the selective synthesis of PMEs by means of the direct esterification of carboxylic acid and panthenol at an equimolar ratio under solvent-free conditions.

### Scope and Evaluation of Different Setups for Scale-Up

A possible industrial application needs a robust synthetic protocol for the preparation of the large scope of panthenol monoesters with carboxylic acids with different alkyl side chain lengths as well as a suitable methodology to scale up the reaction from milligrams to hundreds gram scale. Based on the optimized panthenol:FFA molar ratio at 1:1 (mol/mol), several reaction setups, ultrasound assistance, orbital shaker, rotary evaporator with vacuum, and mechanically stirred reactor with vacuum (see Figure 1B) were tested using nine different FFAs and by changing the process scale by three orders of magnitude. The results obtained for this evaluation are summarized in Table 3.

**Table 3:** Influence of the Alkyl-Chain Length of Carboxylic Acid for the PME Production in Different Setups under Solvent-Free Conditions (Acid:Panthenol, 1:1 mol/mol, 60 °C)

reactor system	carboxylic acid	overall mass g (mmol)	immobilized enzyme (mg/mmol)	conversion (%)	selectivity (%)	PME production (g/h·g N435)
ultrasound assistance^a^	butyric	0.30 (2)	12.5	63	98	4.5
	pentanoic	0.31 (2)	12.5	65	91	4.5
	hexanoic	0.32 (2)	12.5	79	92	5.9
	heptanoic	0.34 (2)	12.5	74	96	6.0
	octanoic	0.35 (2)	12.5	75	96	6.3
	decanoic	0.38 (2)	12.5	79	98	7.4
	lauric	0.41 (2)	12.5	78	99	8.0
	myristic	0.44 (2)	12.5	78	100	8.6
	oleic	0.52 (2)	12.5	76	96	9.1
						
orbital shaking^b^	butyric	2.0 (14.0)	10.0	63	97	4.2
	pentanoic	2.0 (13.2)	10.0	64	90	4.2
	hexanoic	2.0 (12.6)	10.0	67	91	4.6
	heptanoic	2.0 (12.0)	10.0	74	94	5.5
	octanoic	2.0 (11.4)	10.0	69	98	5.6
	decanoic	2.0 (11.0)	10.0	71	99	6.3
	lauric	2.0 (10.2)	10.0	72	100	7.0
	myristic	2.0 (9.6)	10.0	83	100	8.6
	oleic	2.0 (8.0)	10.0	80	100	9.4
						
rotary evaporator + vacuum^c^	hexanoic	40.5 (250)	2.5	87	84	11.0
	heptanoic	42.4 (250)	2.5	89	82	11.6
	octanoic	44.1 (250)	2.5	89	90	13.2
	decanoic	47.9 (250)	2.5	89	83	13.3
	lauric	51.2 (250)	2.5	90	100	17.3
	myristic	54.8 (250)	2.5	92	100	19.2
	oleic	65.1 (250)	2.5	94	100	22.2
						
mechanical stirring + vacuum^d^	hexanoic	324.1 (2000)	2.5	85	94	12.2
	lauric	286.8 (1400)	2.5	83	100	16.0
	lauric	491.6 (2400)	2.5	83	100	16.1

a Reaction time: 1.5 h.

b Reaction time: 2 h.

c Reaction time: 4 h.

d Reaction time: 4 h.

As can be seen in Table 3, regardless of the setup, both the esterification yield and the selectivity for PME, as two key parameters to assess the efficiency of the presented approach, were improved with the increase in the length of the alkyl chain of the acyl donor. The best results (ca. 90% yield and 100% selectivity) were observed for the cases of lauric, myristic, and oleic acids. The longer alkyl side chain of the FFA likely leads to an enhancement of the surfactant activity in the reaction medium, favoring the mass transfer and enhancing the biocatalytic performance. When comparing the low-size systems (*i.e.*, ultrasound assistance and orbital shaker), a similar yield, selectivity, and monoester productivity were obtained. However, both the rotary evaporator and the 500 g reactor with mechanical stirring outstand with the higher yield (*i.e.*, 83–94%), selectivity (*i.e.*, 82–100%), and increase of productivity by an order of magnitude (*i.e.*, 11–22 g ME/h·g N435), as well as the feasibility for further implementation at an industrial scale. This better performance should clearly be related to the shift of the reaction equilibrium toward the esterification product as a result of the continuous elimination of the byproduct water (see Figure 1A) from the reaction medium by the coupled vacuum system. Furthermore, it should be noted that the high-scale reaction systems show an improved esterification yield even after decreasing the amount of the immobilized enzyme 5-fold with respect to the small-scale ones. Taking into account the high viscosity of these reaction media, which ranged from 2500 (panthenol:butyric acid, 1:1 mol/mol) to 81,500 cP (panthenol:myristic acid, 1:1 mol/mol; see Table S1), the mixing of the reaction by either rotation or a stirring paddle results much more efficient to enable appropriate mass transfer within this eutectic mixture formed by the neat substrates. A previous work based on the same biocatalytic esterification approach under shaking conditions also resulted in a moderate esterification yield (*i.e.*, 59%), even though an amount of the immobilized enzyme 12-fold higher was used with respect to this work.^18^ A similar increase in the biocatalytic efficiency at higher-scale esterification reactions was already reported in the selective mono-esterification of xylitol with lauric acid assisted by ultrasounds.^28^ In such a study, the increase from 0.5 to 100 mmol of the overall mass improved not only the yield but also the selectivity and reaction rate, achieving the maximum conversion in just 30 min at 40 °C, being related to a better mixing because of an enhanced transfer of the ultrasonic waves to the core of the mixture. Alternatively, to improve the mass transfer of the reaction species, other authors working on biocatalysis with viscous DESs in lab-on-a-chip flow microreactors^27^ reported the addition of a small amount of water to reduce the viscosity of the reaction system. However, water may have a detrimental effect on the yield of the reversible esterification reactions that becomes more evident in these batch setups. Indeed, the best results in this work were obtained when using an efficient water removal system (*i.e.*, vacuum system), demonstrating the necessity of continuous withdrawal of this byproduct to shift the equilibria toward the synthesis of products.^18^

**Figure 3:**
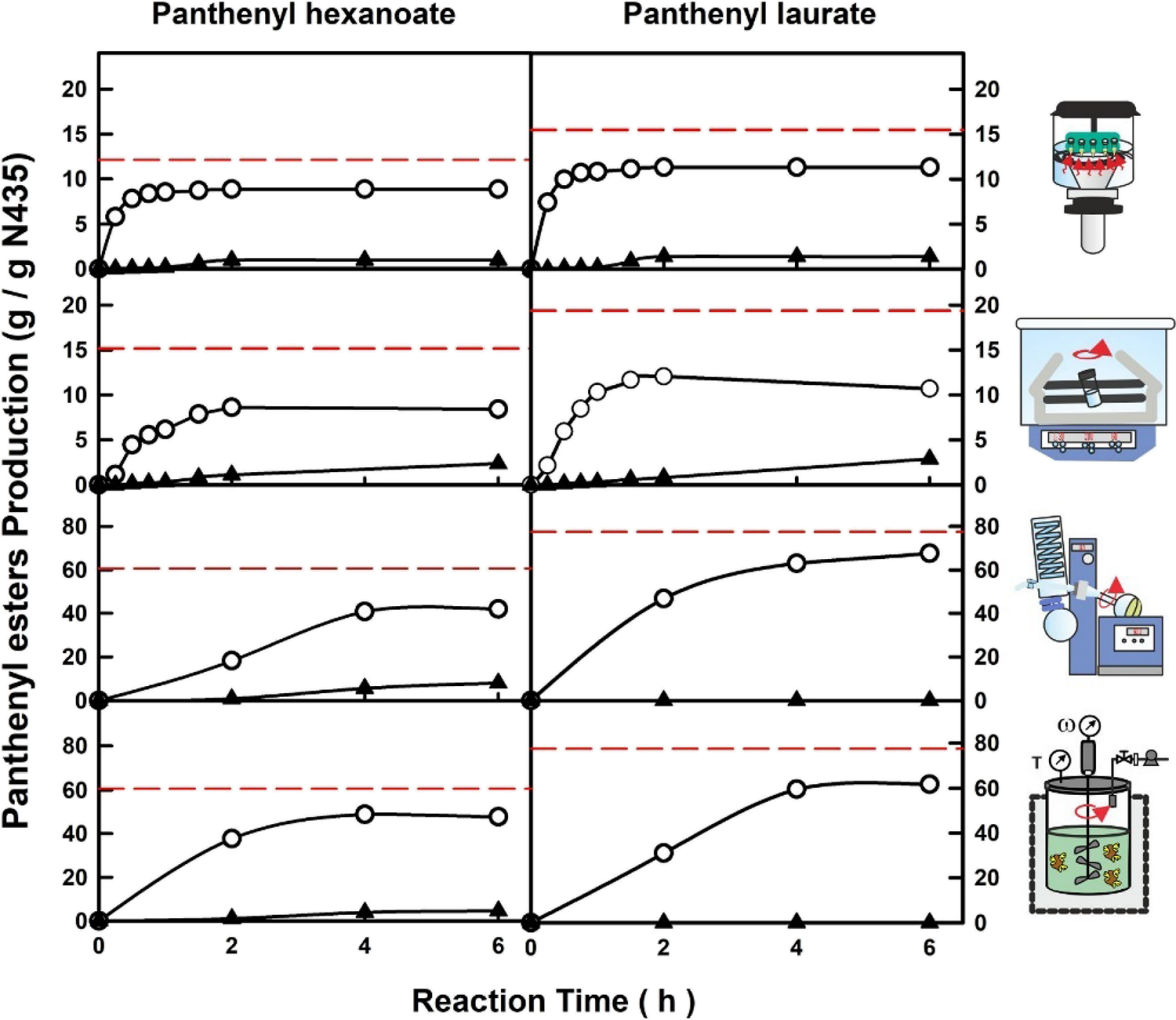
Time-course profiles for the biocatalytic production of panthenyl monoesters (◯) and panthenyl diesters (▲) using hexanoic acid (left) and lauric acid (right) and with different setups: 0.5 g indirect ultrasonication; 2 g orbital shaker; 50 g rotary evaporator with vacuum; 500 g mechanically stirred reactor with vacuum. The profiles show the accumulation of each product per gram of the immobilized biocatalyst (g product/g N435) in the esterification reaction of a mixture of substrates (1:1 mol/mol ratio) according to Table 3. The red dashed line marks the maximum amount of panthenyl monoester to be produced (g ME/g N435) in an ideal condition of 100% yield and selectivity.

Taking the biocatalytic synthesis panthenyl esters of hexanoic and lauric acids as representative examples, Figure 3 depicts the time-course profiles of ester production *per* gram of the immobilized biocatalyst (N435) in each reaction system. In each profile, the red dashed line marks the maximum amount of panthenyl monoesters to be produced (g ME/g N435) in an ideal condition of 100% yield and selectivity.

It can be observed how the maximum level of esterification was reached after 4 h reaction for all cases. It should be noted the higher reaction rate of the biocatalytic process for small-scale reaction setups with respect to the high-scale ones, reaching the maximum level of esterification at 1.5 and 2 h for ultrasound assistance and orbital shaking setups, respectively, due to their higher concentration of biocatalysts (see Figure 3). It should be taken into account that reactor systems with poor mass transfer of substrates limit the entry of bare panthenol molecules to the enzyme microenvironments, favoring the multi-esterification, which becomes more evident in the orbital shaking setup. In the same way, the improved performance showed by high-scale reactor systems, near the ideal ME production/g N435 and selectivity parameters, should be related to the better mass transfer of the substrate viscous mixture, together with the continuous water removal.

**Figure 4:**
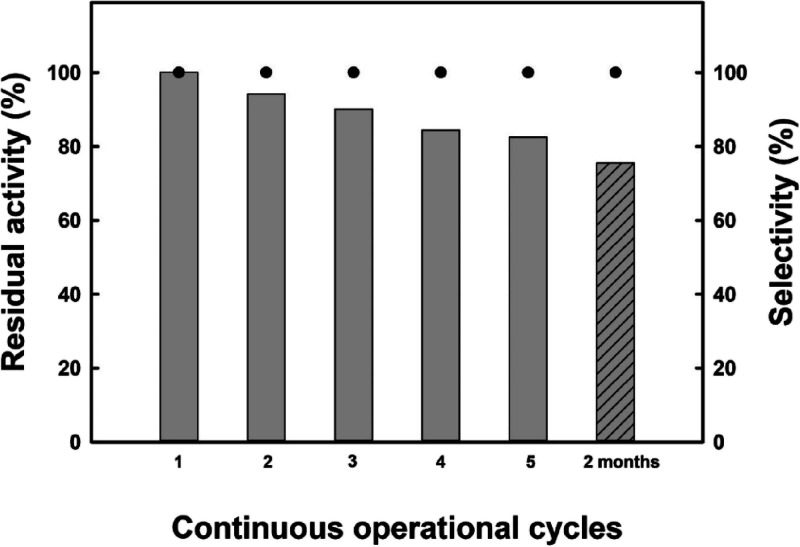
Operational stability of N435 in the synthesis of panthenyl monolaurate by direct esterification of lauric acid with panthenol (1: 1 mol/mol ratio) as a DES-based reaction medium at 60 °C. Reactions performed in a rotary evaporator, 250 mmol overall mass, 60 °C, 140 rpm, 8 h. The dashed bar (right) shows the activity after recovery and storage of the enzyme for 2 months at 8 °C.

Furthermore, the role of the setup in the stability of the biocatalysts is another key criterion in the optimization of the reaction conditions for high-scale synthesis, regarding the further reusability of the immobilized biocatalyst and costs of the process.^15,18^ As can be seen in Figure 4, the immobilized enzyme showed excellent operational stability toward reuse for the high-scale rotary evaporator system for five consecutive operation cycles and even after 2 months of storage at 4 °C. This maintenance of the biocatalytic activity may be related to the stabilization power of panthenol as a polyol. The suitability of polyols (*e.g*., xylitol, sorbitol, etc.)^28,38^ and DES (*e.g*., urea:choline chloride mixture),^39^ as protecting agents of biocatalysts against deactivation, has been widely reported, being attributed to a hydrogen binding net interacting with proteins that maintain the solvophobicity, as a key parameter for supporting the native conformation of the enzyme. The variations in the residual activity were due to the mass loss of the immobilized enzyme in the process of manual recovery between assays, considering the reduced amount of N435 used (2.5 mg/mmol overall mass) and the highly viscous reaction media (see Table S1). Even then, the residual activity is over 80% in all cycles and the selectivity is not compromised by this loss, which emphasizes the strength and appropriateness of this biocatalytic strategy for industrial implementation.

### Environmental Impact of the Technology

Environmental impact is a key criterion to be considered for any synthetic process aiming to be developed at the industrial level. In this work, a comparative analysis among reported strategies for panthenol esterification was carried out, taking into account all inherent characteristics of each process related to sustainability (*i.e.*, organic solvent, organic catalysis *versus* biocatalysis) listed in Table 4.

**Table 4:** Comparative Analysis of Different Reported Strategies for Producing Panthenyl Ester Products

entry	acyl donor (mmol)	molar ratio^a^	solvent (mL)	catalyst (g)	temp (°C)	time (h)	yield (%)	products (% Sel)^b^	productivity (g/L·h)	PMI (S&R; Sv)^c^	*E*	EcoScale	ref
1	acetic anhydride (4.4)	1:8	none	DMAP (2.0)	80	9	78	panthenyl triacetate (100%)	33	3.8 (3.8; 0.0)	3.2	68	(11)
2	methyl acrylate (10,000)	1:10	acetone (200)	N435 (10.0)	40	2.5	96	panthenyl monoacrylate (99.0%) and diacrylate (1.0%)	3.2	8.1 (4.3; 3.8)	7.1	69	(13)
3	isopropyl acetate (30)	1:2	ACN (100)	N435 (1.0)	30	20	100	panthenyl diacetate (100%)	2.1	19.4 (1.4; 18.0)	19.5	73	(12)
4	lauric acid (125)	1:1	none	N435 (0.6)	60	4	90	panthenyl monolaurate (100%)	217.6	1.2 (1.2; 0.0)	0.2	91.8	this work

a Molar ratio of panthenol:acyl donor.

b Selectivity.

c S&R, substrates and reagents; Sv, solvents; DMAP, dimethylaminopyridine; ACN, acetonitrile.

Since the esterification of the hydrophobic fatty acids with the hydrophilic panthenol is not an easy task, different strategies have been reported to improve the mutual miscibility with the acyl donor by using different solvents (*i.e.*, acetone^13^ or ACN^12^) or acyl donor derivatives that reduce their initial hydrophobicity (*i.e.*, acetic anhydride,^11^ methyl acrylate,^13^ or isopropyl acetate^12^). Nevertheless, the use of these substrates means additional unsustainable steps of previous modification and the presence of non-desired byproducts as results of the transesterification (*i.e.*, acetic acid and sodium acetate^11^ or methanol^13^). Overall, any of those strategies involves the necessity of further purification steps to remove any traces of solvents or byproducts. In addition, it should be noted the high molar ratio of substrates used (entries 1 and 2), the low selectivity in the panthenol mono-esterification (entries 1 and 3), or the high excess of (bio)catalyst (entries 1 and 3) used. In contrast, the direct esterification approach between panthenol and natural fatty acids solventless (entry 4) avoids downstream processing since non-reacted substrates are usual ingredients in cosmetic formulations, and water is the only byproduct. In addition, this solvent-free protocol not only permits the highly selective mono-esterification of panthenol with highly hydrophobic acyl donors but also enables the higher productivity of selective panthenyl monoesters (217 g/L·h) with the best economy of substrates and the biocatalyst.

However, to assess the sustainability of the overall approach of each synthetic strategy listed in Table 4, several green metrics parameters (*i.e.*, process mass intensification, E-factor, TCR, and EcoScale) have been used. The process mass intensification (PMI) parameter outstands as the best tool to determine the material efficiency in a process, facilitating the analysis of the input–output mass balance (see Table 2).^40^ Thus, the PMI correlates with RME and the E-factor.^41^

The ACS PMI Calculator^29^ permits the easy determination of this metric, as well as discerning the contributions of reagents and solvents during the work-up and purification steps to the PMI value (see calculation data in Table S2). Therefore, any step of the approach that contributes to waste generation can be identified for further improvement of the overall process. According to Table 4, entries 2 and 3 show the higher PMI values as results of the contribution of reagents/solvents as the main source of waste. On the contrary, solvent-free strategies (entries 1 and 4) show lower PMI values, which only account for non-reacted substrates and reagents. The best PMI and E-factor scores (entry 4) also point out the higher suitability of the methodology with respect to the previous ones.

In agreement with the PMI and E-factor, the total carbon dioxide release (TCR) is another recent green metric parameter that provides the amount of CO_2_ produced per kg of the product by considering all the steps involved in the process, including purification steps and wastewater treatments. This TCR parameter can be calculated according to the equation proposed by Onken et al.^42^ (see Table 2), where the worst scenario of waste treatment, such as the total incineration, is considered. Thus, the TCR offers a frame to compare different strategies at the benchmark scale.^43^ However, this parameter cannot be determined for entries 1–3 due to the absence of information about downstream processes. For the strategy here presented, as a purification step is not required, wastes and wastewater are not produced, so the TCR parameter should be equal to zero. However, by considering the PMI value of the organic compounds (Table 4, entry 4), the TCR parameter results in a value of 2.8, being very close to “waste-free” systems.^43^

However, the PMI, E-factor, and TCR metrics do not cover issues related to the environmental and safety-hazard risks, which are considered by the EcoScale.^30^ The EcoScale value comprises ecological and economical aspects in the evaluation of a reaction and may be very useful to perform a preliminary study of the life cycle assessment of a process.^44,45^ This tool focuses on the environmental aspects of the reagents used, the methodology implemented, and the yield of the process. To perform the analysis, the EcoScale evaluates all materials involved in the reaction according to six categories with special emphasis on their toxicity and price, the reaction conditions (temperature and reaction time), synthesis procedure, work-up/purification processes, and yield of the isolated product, introducing penalties that decrease an initial value of 100% that would correspond to the highest sustainability (see calculation data in Table S3). This tool makes a clear feature of the weaknesses of the strategies pointing to the low yield and materials safety as the main drawbacks. According to Table 4, the use of derivatized substrates like acetic anhydride, methyl acrylate, and isopropyl acetate as well as hazardous solvents (entries 1–3) is penalized with the lower EcoScale values not only because of their unsustainable nature but also due to the need of additional work-up and purification steps. On the other hand, this tool rates with the highest value the strategy 4 (91.8) based on the use of natural substrates, the absence of solvent, and the high yield.

Notwithstanding, it is important to note the weaknesses of the EcoScale analysis. For example, this tool only considers the yield, but no penalties on atom economy are set.^38^ This situation leads to misestimations where non-stoichiometric approaches with a high excess of a substrate can obtain low penalties. Also, the waste products are ignored despite the impact of their accumulation in the environment, and a rough distinction in the conditions of temperature and reaction time is made. Because of this, different modifications have been made to this tool. For instance, Boehringer Ingelheim made an adaptation of this tool for the industry that provides for a finer adjustment of different parameters, especially those related to the reaction temperature and reaction time.^46^

Thus, it seems evident that meanwhile a single green metric may show some deficiencies, the combined use of the selected metrics can give an accurate overview of the sustainability and suitability of a process for further industrial implementation. The results obtained in this analysis point to entry 4 as the more sustainable approach due to the use of natural substrates and the absence of solvents. Even more, these scores highlight how the sustainability of this approach relies on the higher atom economy, based on an equimolar ratio of substrates and the improved productivity and selectivity, that results in the lower PMI and E-factor and the higher EcoScale values.

It should be noted that these metrics have not considered the amount of (bio)catalysts used due to their recovery and reuse. However, it is important to highlight that the strategy here developed significantly reduces the amount of the biocatalyst, which together with its further reuse affords a higher utilization and cost reduction.

## Conclusions

The biocatalytic synthesis of panthenyl monoesters in solvent-free systems based on eutectic mixtures between the neat substrates is a suitable strategy that can be easily implemented at higher scales of production. The optimization of the operation conditions and the excellent performance of the biocatalyst improve key reaction parameters like the reduction of the amount of the biocatalyst used, atom economy, selectivity, process simplification, and significant waste reduction, which are evidenced by the scores obtained in the different metrics used, PMI, E-factor, and EcoScale. Beyond the sustainable issues, the optimized conditions also improve the economy of the process with respect to other synthetic strategies previously reported, as a result of the absence of solvents and the use of natural substrates, which permits a straightforward application as well as the easy recovery and reuse of the biocatalyst for several operational cycles.

As a result, the implementation of this approach provides the clean and sustainable synthesis of a wide range of panthenyl monoesters with different lengths in the acyl chain and chemical–physical properties and with good productivity to fit with the demands of cosmetic formulations. Once again, the sustainable path for synthesizing bioactive compounds has been demonstrated by the proper design of reaction media, pointing out the possible implementation at an industrial scale.

## Supporting Information Available

^13^C and ^1^H NMR analyses of the panthenol esterification with hexanoic acid; table with the physical–chemical properties of the reaction mixtures of panthenol monoesters; tables with the numeric calculations for PMI and EcoScale green metrics parameters (PDF)

## Abbreviations

AE: atom economy
DESs: deep eutectic solvents
DMAP: dimethylaminopyridine
E-factor: environmental factor
FFA: free fatty acid
MRP: material recovery parameter
RME: reaction mass efficiency
PMI: process mass intensity
SF: stoichiometric factor
TCR: total carbon release
