# Supplementary material for: Sustainable Setups for the Biocatalytic Production and Scale-Up of Panthenyl Monoacyl Esters under Solvent-Free Conditions
Source: ACS Sustain Chem Eng. 2023 Mar 22;11(14):5737–47. doi: 10.1021/acssuschemeng.3c00266 (PMC10091472; doi:10.1021/acssuschemeng.3c00266)
Supplement: Supplementary file 1 — sc3c00266_si_001.pdf [file sc3c00266_si_001.pdf]

Supporting Information

Sustainable set-ups for the biocatalytic production and scale-up of panthenyl monoacyl esters under solvent-free conditions.

Susana Nieto[a], Juana M. Bernal,[a] Rocio Villa,[a,b], Eduardo Garcia-Verdugo,[c] Antonio Donaire,[d] Pedro Lozano [a].*

[a] Departamento de Bioquimica y Biologia Molecular B e Inmunologia. Facultad de Quimica, Campus de Espinardo, Universidad de Murcia, E-30100 Murcia, Spain

[b] Department of Biotechnology, Delft University of Technology, Delft, The Netherlands

[c] Departamento de Quimica Organica e Inorganica, Universidad Jaime I, E-12071, Castellon, Spain

[d] Departamento de Quimica Inorganica. Facultad de Quimica, Campus de Espinardo, Universidad de Murcia, E-30100 Murcia, Spain

* Corresponding authors

Susana Nieto. Departamento de Bioquimica y Biologia Molecular B e Inmunologia. Facultad de Quimica, Campus de Espinardo, Universidad de Murcia, E-30100 Murcia, Spain. ORCID: 0000-0003-3026-7876. Phone: +34 868 889437. E-mail: susanani@um.es

Pedro Lozano

Departamento de Bioquimica y Biologia Molecular B e Inmunologia. Facultad de Quimica, Campus de Espinardo, Universidad de Murcia, E-30100 Murcia, Spain. ORCID: 0000-0001-6043-3893. Phone: +34 868 887392. E-mail: plozanor@um.es

Number of pages: 7

Number of figures: 2

Number of tables: 3

1. NMR data

The biocatalytic esterification of hexanoic acid with panthenol under solvent-free conditions in the rotary evaporator (6 h, 60 °C) was taken as the model reaction for the NMR analysis. An aliquot of 50 μL from the reaction mixture was dissolved in DMSO-δ_6 to a final volume of 500 μL and analyzed by a 600 MHz NMR Brucker spectrometer.

^{13}C NMR spectrum

The expansion of the ^{13}C NMR spectrum corresponding to the region of the carbons bound to OH is shown in Figure SI-1 (55-80 ppm) for the panthenol molecule (A) and the panthenyl hexanoate ester (B). This spectrum allows us to quantify the ester bound for each specific hydroxyl group. As observed the main ester results from the esterification of the 3’ hydroxyl carbon.

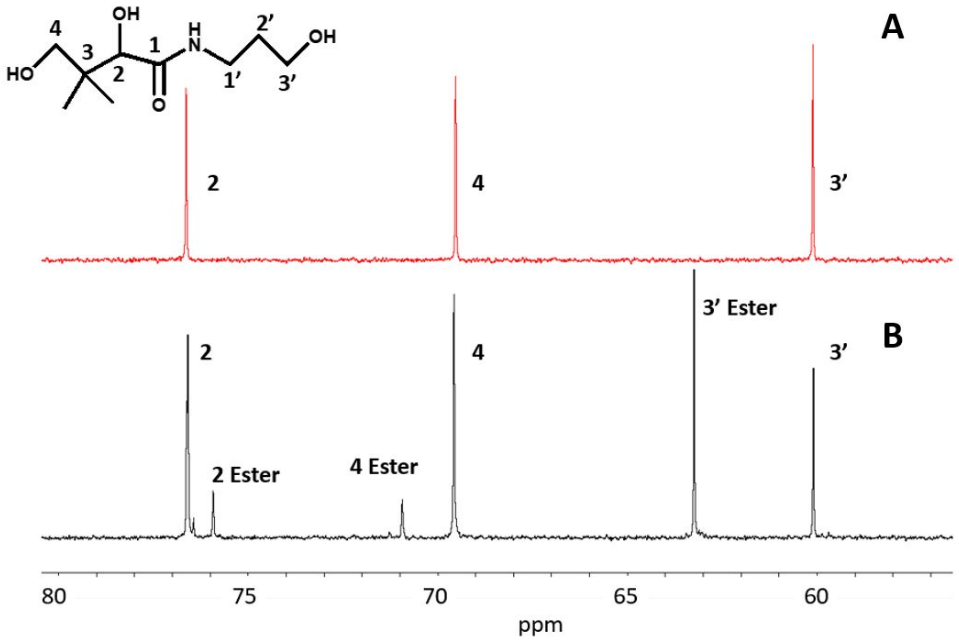

Figure S1. Expansion of the ^{13}C NMR spectrum corresponding to the region of the carbons bound to OH in the panthenol molecules (A) and for the panthenyl hexanoate (B). The assignments of the signals are given according to the shown formula. According to the areas of these signals, the hydroxyl group of the C3 hydroxypropyl position of panthenol is the preferable position of esterification accounting for 68% of the ester products, followed by C2, (19%) and C4 (13%).

^{1}H NMR and DOSY spectrum

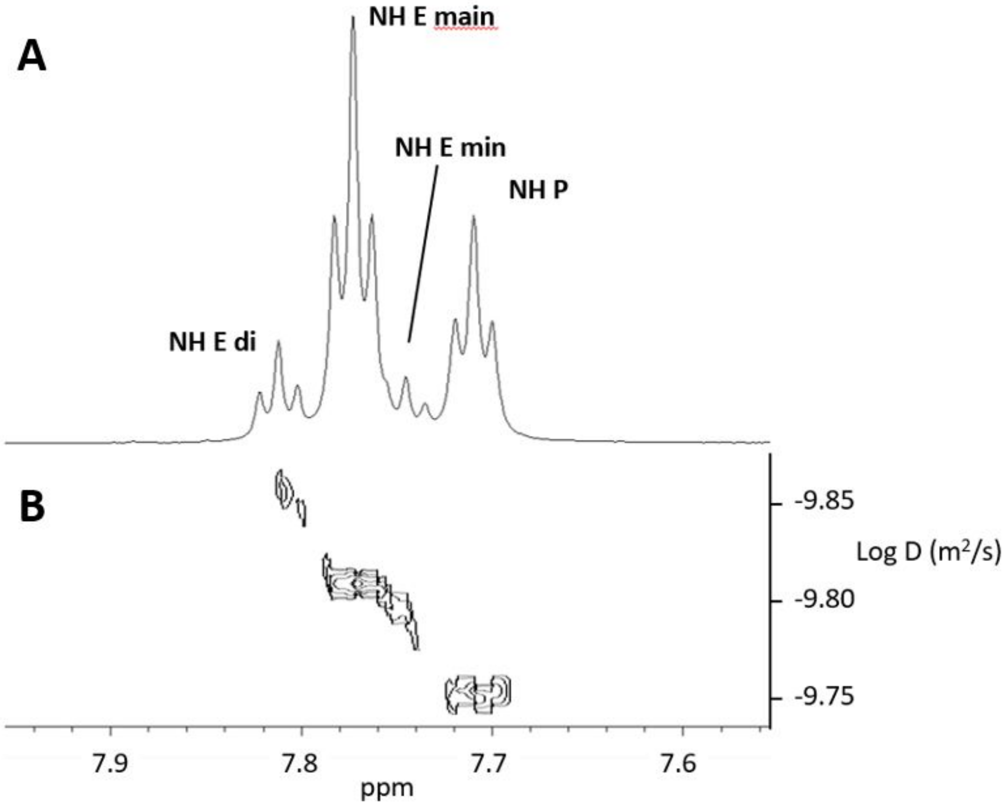

Figure S2. A. ^{1}H NMR spectrum corresponding to the NH signal of the panthenol moiety for the sample corresponding to the reaction between panthenol and hexanoic acid. Four signals are observed, namely: NH P, for the panthenol non-reacted molecule (7.71 ppm); NH E min, for a minor ester species (7.75 ppm); NH E main, for the major ester species (7.77 ppm); and NH E di, for the diester species (7.81 ppm). B. Diffusion 2D experiment (DOSY spectrum) corresponding to the same region that confirms the assignment. Three species corresponding to three moieties with three different translational diffusion coefficients (*i.e.*, with different molecular weights) are observed corresponding, from the highest to lowest coefficients, to: panthenol, monoesters and diester species (from the bottom to the top of the 2D spectrum). From the intensity of the NH at the highest frequency (NH E di) in the ^{1}H NMR spectrum the ratio of the monoesters and diesters can be calculated resulting in 84.5% and 15.5 % respectively, in agreement with the data of Table 3 in the manuscript.

2. Determination of the physical-chemical properties of the products.

The analyses were performed with the products synthesized using the rotary evaporator set-up. The pH was evaluated using a pH-meter (SensION TM+, Germany), and the

viscosities were measured using a P-Selecta Viscometer (ST-2020R, Spain), with spindles R4* and R7** depending on the viscosity value and an angular speed of 20 rpm. The dry residue was quantified with thermobalance equipment (Kern DBS, Germany), using a constant temperature of 120°C.

Table S1. Physical-chemical characterization of panthenyl esters.

Ester alkyl chain	pH	Viscosity (20°C, cP)	Dry Residue (%)
C6	6.6	3798*	99.3
C7	7.0	2532*	98.3
C8	6.8	3092*	99.0
C10	6.7	14125**	99.1
C12	6.7	59164**	98.9
C14	7.0	81408**	98.1
C18Δ^9	7.0	1815*	98.8

*Spindle R4

**Spindle R7

All samples showed a pH close to 7.0 and a water content lower than 2%. According to these data, the synthesized products have the right properties for their incorporation into hydrophobic formulas of commodities.

The length and structure of the alkyl chain of the FFA determine major differences in the viscosity of the final ester product ranging from 1815 – 81408 cP. All reaction mixtures were liquid at room temperature but those with the higher viscosity, the panthenyl ester products with C12 and C14 length alkyl chains.

3. Metrics calculation

The ACS PMI Calculator and the EcoScale tool were used to determine the sustainability and eco-economical aspects of synthesis reactions. Tables SI-2 and 3 show the parameters used to calculate these metrics for entries 1-4 in Table 3 in the manuscript.

Table S2. Parameters introduced in the ACS PMI Calculator.

Entry	1	2	3	5
Molar Yield %	80%	96 %	100 %	89.6 %
Product (g)	(112.4)	(248.7)	(4.3)	(43.4)
Substrates (g)	Panthenol (87)	Panthenol (205)	Panthenol (3.1)	Panthenol (25.7)
Reagents (g)	Acetic anhydride (336.9) DMAP (2.0)	Methyl acrylate (861) Methoxyphenol (0.05)	Isopropyl acetate (3.6)	Lauric acid (25.0)
Solvents (g)	0	Acetone (158) Isopropanol (785)	Acetonitrile (78.1)	0
Aqueous	0	0	0	0
Step PMI	**3.8**	**8.1**	**19.5**	**1.2**
Step PMI Substrates and Reagents	3.8	4.3	1.5	1.2
Step PMI Solvents	0	3.8	18	0.0

Table S3. EcoSscale analysis of the ecological and economical parameters of selected strategies for panthenol acylation. The table shows the assigned penalties according to the reaction conditions. The last row reveals the final EcoScale score.

**Entry**	**1**	**2**	**3**	**5**
Yield	-10	-2	0	-5.2
Price availability	-15	-5	-3	0
Safety*				
Acetonitrile (F, T)			-10	
Acetone (F)		-5		
Methyl acrylate (F)		-5		
Isopropanol (F)		-5		
Isopropyl acetate (F)			-5	
Technical setup				
Common setup		0	0	0
Instruments for controlled addition of chemicals	-1			
Temperature-Time				
Heating > 1h	-3	-3	-3	-3
Work up and purification				
Adding solvent	0			
Simple filtration	0			0
Liquid-liquid extraction or washing		-3	-3	
Distillation	-3	-3	-3	0
Cooling				
**EcoScale score**	**68.0**	**69.0**	**73.0**	**91.8**

*F: Highly Flammable (5 points penalty); T: Toxic (5 points penalty).
